# Design and Development of a Digital Weight Management Intervention (ToDAy): Qualitative Study

**DOI:** 10.2196/17919

**Published:** 2020-09-09

**Authors:** Charlene L Shoneye, Barbara Mullan, Andrea Begley, Christina M Pollard, Jonine Jancey, Deborah A Kerr

**Affiliations:** 1 School of Public Health Curtin University Perth, Western Australia Australia; 2 Health Psychology & Behavioural Medicine Research Group School of Psychology Curtin University Perth, Western Australia Australia; 3 East Metropolitan Health Service Perth, Western Australia Australia; 4 Collaboration for Evidence Research & Impact in Public Health School of Public Health Curtin University Perth, Western Australia Australia

**Keywords:** obesity, diet, physical activity, sedentary behavior, digital behavioral interventions, health behavior, wearable activity monitor, health, mobile food record, clinical trial, focus group, qualitative research, mobile phone

## Abstract

**Background:**

The Tailored Diet and Activity (ToDAy) study aims to build on the campaign by adding a digital intervention with the potential to provide wide-reaching, cost-effective weight management support.

**Objective:**

The ToDAy study aims to build a tailored intervention using mobile technology to improve diet and physical activity behaviours in adults with overweight and obesity. The main objectives were to identify behavior change techniques for diet and physical activity (PA) change for weight loss and explore preferences for digital intervention features that would be effective in changing diet and PA behaviors.

**Methods:**

This qualitative study uses the principles of a person-based approach to intervention development; the behavioral intervention technology framework; and the capability, opportunity, motivation, and behavior (COM-B) framework. Focus groups and telephone interviews were conducted with 56 adults in Western Australia. Open-ended questions and example intervention features were used to explore the usability and acceptability of the self-monitoring tools, knowledge about effective weight-loss strategies, and acceptability of tailored feedback. Findings from the focus groups and interviews were analyzed using thematic analysis.

**Results:**

Qualitative findings revealed an awareness of key public health messages but a lack of confidence in how to perform these behaviors to help manage their weight. A total of 4 major themes were identified and mapped to the domains of the COM-B framework: (1) misinformation, (2) environmental support, (3) social norms, and (4) confidence.

**Conclusions:**

This study explores users’ capability, opportunity, and motivation to perform the target behaviors for weight loss. The findings suggested that a digital weight management intervention using a mobile food record and activity trackers to inform tailored feedback may be acceptable and feasible. Participants expressed a preference for simple expert advice, digital self-monitoring tools, and visual feedback.

**International Registered Report Identifier (IRRID):**

RR2-10.2196/12782

## Introduction

### Background

Excess weight has overtaken smoking as the leading cause of noncommunicable disease in Australia, with 7 out of 10 males and almost 6 out of 10 females living with overweight or obesity [1]. The causes of this are multifaceted [2] but, at a personal level, poor diet and inactivity are major contributors. In Australia, excessive intake of alcohol, sugar-sweetened beverages (SSBs), and *discretionary foods* (foods considered to be of little nutritional value; often high in saturated fats, added sugar, and salt; and alcohol or *junk* foods) are observed across all age groups [3]. Mass media campaigns, targeting healthy weight, positively influence knowledge and awareness with modest impacts on behavior [4-7]. LiveLighter is a Western Australian public health education and social marketing campaign that aims to encourage people to eat well, be physically active, and maintain a healthy weight. The campaign engages with the community through paid and unpaid social media, web-based and printed resources, and retailers. Campaign messages include graphic images of *toxic* fat, followed by messages with single actions to reduce the risk of weight gain, for example, by avoiding SSBs or junk food [8]. The advertisements also direct people to a campaign website where there is an option to enroll on the web to access a meal planner, recipes, and weight-monitoring tools and to receive update emails. The Tailored Diet and Activity (ToDAy) study aims to build a digital intervention that provides individualized tailored feedback on dietary and activity behaviors.

In Australia, the evaluation of the LiveLighter mass media campaign targeting sugary drinks indicates a high campaign recall and modest reductions in SSBs [4,9-11].

However, >60% of adults in Australia do not usually consume SSBs and may disregard the campaign, even if they stand to benefit from some of the other elements. A growing body of evidence supports the notion that information is best tailored specifically to the unique characteristics and behaviors of an individual [12-17], with significant effects reported for nutrition and PA.

Australian clinical guidelines for weight management recommend a multidisciplinary team of health professionals using specific behavior change techniques (BCTs) applied for a minimum of 12 months [18]. High attrition rates, low availability of trained health professionals, and logistical issues are among the commonly reported barriers to putting this in practice [19]. Digital interventions that combine clinical and tailored content with the reach of mass media could be a cost-effective solution [20].

### Developing Digital Interventions

The process of developing a digital intervention requires integrating the behavior change theory with intervention features that are engaging and acceptable to the target group. Evidence from other digital behaviour change interventions suggest an iterative and multidisciplinary approach that includes a qualitative investigation with the end user before implementation [14,21,22]. Specifically, the person-centered approach recommends using qualitative research to explore and test intervention features with the target group [23]. This allows researchers to adapt the intervention features based on the preferences and needs of the user. A BCT is the *active ingredient* or intervention component that changes the desired behavior [24]. For example, BCT 2.3 is self-monitoring of behavior, the most commonly used BCT in effective diet and PA interventions [25]. Evidence suggests that self-monitoring works through behavioral regulation; for example, dietary self-monitoring increases the awareness of food choice, portion sizes, and improving diet quality [25,26,27]. Exploring digital interventions for weight management that combine the reach of mass media campaigns with tailored and clinical support could be a cost-effective and practical approach.

Focus groups and interviews are commonly used to generate discussion and explore participants’ experiences as well as their needs, knowledge, and preferences [28,29]. However, weight stigma inhibits participation and the willingness to share personal beliefs [29,30]. One strategy to address this is to show participants hypothetical scenarios and ask them to provide advice for weight loss [28,29]. Another strategy to address this is to use hypothetical scenarios where information about a person’s PA levels or images of their meals is shown and participants are asked to provide advice on weight loss. To date, this unique approach has not been undertaken in this population.

Although men are more likely to be living with excess weight and experience health-related illnesses, they are less likely to participate in lifestyle interventions, making up only about 20% of participants [31]. Studies aiming to reduce this gender imbalance have reported that self-monitoring technology, including mobile apps and wearable devices, are great incentives to engage men in weight management [32,33]. To date, few weight management studies have included the views and experiences of men aged >25 years [34]. This study aims to address this shortcoming by purposely sampling an equal number of male and female participants.

This study aims to describe the qualitative study and iterative process used to develop ToDAy, a digital, tailored, weight management intervention. A full description of the aims of the 12-month intervention and the protocol has been published elsewhere [35]. The objectives of this study were to (1) identify BCTs for dietary and PA changes concerning weight loss and (2) explore preferences for digital intervention features that would be effective in changing diet and PA behaviors.

## Methods

### Study Design

The methodological approach used in this research was a general inductive, qualitative approach [36]. The Consolidated Criteria for Reporting Qualitative Research for interviews and focus groups were used to ensure rigor in the presentation of the findings [37]. ToDAy will incorporate learnings from an earlier trial where a mobile food recording app (mFR) was successfully used to assess dietary intake and provide tailored feedback on fruit, vegetable, and junk food intake in young adults [38,39].

Open-ended questions and example intervention features were used to explore the usability and acceptability of the self-monitoring tools, knowledge about effective weight loss strategies, and acceptability of the tailored feedback.

We used an iterative intervention development process applying the behavioral intervention technology (BIT) theory [40,41] and the person-based approach [23]. An overview of the 3 stages of intervention development is provided below.

### Stage 1

To derive the measurable and clinically significant behavioral changes that could be expected from the intervention, we reviewed the recent literature and evidence-based guidelines from the Australian scientific authoritative bodies. These included the National Health and Medical Research Council’s clinical guidelines for weight management [18], the Australian Dietary Guidelines [42], and the Australian government’s PA guidelines [43]. We determined the clinically significant target behaviors for the intervention as follows:

Dietary:

1. Daily dietary energy reduction of 2000 kJ.
2. Avoiding or limiting energy-dense nutrient-poor (EDNP) foods, SSBs, and alcohol.
3. Eating less at meals or additional snacks (except fruits and vegetables).
4. Eating less often [18,42].

PA:

1. Daily step count ≥10,000.
2. ≥30 active minutes (spent in moderate-to-vigorous PA).
3. ≥250 steps per hour [43].

Weight loss:

1. 5% reduction in body weight [18].

Target behaviors: The ToDAy study investigated whether a digital, tailored intervention can improve diet and PA behaviors in adults with overweight or obesity. A total of 16 health professionals with expertise in dietetics, PA, health promotion, and community engagement were consulted in a series of 5 workshops and meetings to explore the target behaviors needed to achieve the clinical aims and where, when, why, and who they occur with [45].

### Stage 2

The research team developed a user-friendly script for the focus groups and interviews with a male and female consumer representative [46]. Focus groups and interviews were conducted with volunteers to explore the acceptability of the selected BCTs and their preferences for digital intervention features. The findings of these focus groups are presented in this paper.

### Stage 3

Target behaviors were mapped to possible intervention features by the research team with reference to previous research [47,48] and following guidelines for developing complex behavior change interventions [49,50]. Focus groups and interviews were followed by a review of intervention features by the research team. This was repeated in a cyclical manner to allow continued user involvement in the design and development of the final intervention.

Approval for the study was granted by the Curtin Human Research Ethics Committee (HR E2016-0271). All participants agreed to an audio recording of their focus group or interview and provided informed consent. All data were collected between October and November 2016 in Western Australia (spring).

#### Theoretical Frameworks

Several guidelines exist for the development and assessment of evidence-based apps and web-based interventions [50-52]. As this intervention uses a combination of digital tools, that is, an mFR, a wearable PA tracker, text messages, and emails, a combination of theoretical approaches and guidelines was drawn upon. The BIT framework was used to identify the technology and procedures for delivering clinical aims and BCTs (objective 2) [40]. The models help to identify clinical aims and link these with suitable intervention features for testing with the user (Table 1).

The capability, opportunity, motivation, and behavior (COM-B) model was then used to guide the selection of intervention features and strategies such as self-monitoring, goal setting, motivation enhancement, and feedback on performance (objective 1) [45]. The COM-B model aims to specify behavioral targets and support psychological theories when developing interventions [45]. The COM-B model states that 3 factors are needed to change behavior: capability (C), opportunity (O), and motivation (M). According to this model, performing a behavior (B) first requires individuals to be capable (C) or have physical and mental abilities (eg, nutrition knowledge, cooking skills). Following this is opportunity, which includes both practical and social aspects (eg, access to healthy food that is culturally acceptable and within social norms). Finally, motivation includes automatic drivers like habits as well as beliefs, plans, and impulses. Table 1 illustrates the steps in the development process—why, how, what, and where? The *what* includes the BCT and associated taxonomy number to identify each BCT from the Behavior Change Technique Taxonomy v1 (BCTTv1) [53].

**Table 1:** Relationship among clinical aims, behavior change techniques, and intervention features (technology).

Why? Clinical aim or population health focus	How? Action	What: behavior change techniques^a^ [53]	Where: potential intervention features tested in qualitative research
Reduce BMI by 5% [18]	Reduce energy intake by 2000 kJ per day and increase PA^b^ (10,000 steps)	Provide information on the consequences (5.1), goal setting (behavior and outcome; 1.1, 1.3), and review of behavior goals (1.5)	Tailored feedback, weight tracker, PA tracker
Reduce EDNP^c^ foods [42]	Increase awareness of EDNP intake	Goal setting (behavior and outcome; 1.1, 1.3), review of behavior goals (1.5), provide feedback on behavior (2.2), and social comparison (6.2)	Mobile food record; tailored feedback and tailored education; app alerts, eg, Have you had any snacks today?
Reduce SSBs^d^ [42]	Increase awareness of energy in SSBs and intake	Self-monitoring of behavior (2.3), goal setting (1.1), barrier identification, provide feedback on behavior (2.2), and social comparison (6.2)	Mobile food record tailored feedback and tailored education
Increase fruit and vegetable consumption [42]	Increase awareness of current intake	Self-monitoring of behavior (2.3), discrepancy between current behavior and recommendations (1.6), action planning (1.4), problem solving (1.2), and instruction on how to perform behavior (4.1)	Mobile food record tailored feedback and tailored education
Reduce alcohol intake [54]	Increase awareness of current intake	Information on health consequences (5.1), motivational interviewing, and self-monitoring of behavior (2.3)	App alerts, eg, How confident are you about having an alcohol-free dinner tomorrow night?

^a^Behavior change technique and associated taxonomy from the Behavior Change Technique Taxonomy v1 (BCTTv1) [53].

^b^PA: physical activity.

^c^EDNP: energy-dense nutrient-poor.

^d^SSBs: sugar-sweetened beverages.

#### Recruitment

Recruitment was specific and purposeful [55], aiming for a similar number of males and females and including people with overweight or obesity who had some experience of the LiveLighter campaign [8]. A single recruitment email was sent to 20,000 adults who had registered with the LiveLighter website in October 2016. The email was sent to the entire mailing list of LiveLighter members, inviting them to take part in the study by clicking on a study web link where participants completed web-based consent and screening. The website was closed after 2 days as 245 respondents had completed the screening questionnaire. The respondents who met the criteria were sent further details on how to participate in a focus group or interview. There were 145 eligible participants, who were >18 years and had a BMI over 25 kg/m^2^. The time, date, and location of the focus groups were sent to the eligible participants to allow them to choose the most convenient time. As 85% of the sample were women, additional recruitment strategies were employed to encourage male participants, such as offering a one-to-one telephone call and men-only focus groups. When these additional approaches were not successful, a workplace with a high proportion of males was contacted and an onsite focus group was arranged, with 14 men in attendance.

### Script Development

The topics covered in the focus groups and interviews were informed by the literature as important features for weight loss interventions and included self-monitoring of diet and PA behavior, feedback on performance, reducing intake of discretionary food drink and alcohol, reducing sedentary time, and increasing steps per day. The script was pilot tested with researchers at Curtin University and 2 consumer representatives where feedback on clarity was provided. Focus groups were conducted in community settings, community centers, place of work, and Cancer Council WA meeting rooms. For each session, the script was accompanied by a visual presentation of example images and draft intervention features. Multimedia Appendix 1 contains the basic script used for focus groups and interviews.

A semistructured focus group and interview guide with open-ended questions were developed, which allowed an iterative, person-centered data collection process [23,56]. As a result, a variation of the script was used in each session. For example, “the last group suggested the dietary feedback include their food images so they can see where the junk food came from. What do you think of this idea? Have a look at this example. Is there anything you would change?”

### User Preferences

Preferences for digital intervention features explored willingness to use the digital self-monitoring tools as well as the frequency and duration of self-monitoring. Preferences for digital feedback explored the format, frequency, length, and content of the tailored diet and PA feedback. With regard to digital content to address the target behaviors, participants were asked to suggest helpful advice and potential barriers to changes for a particular behavior, for example, “What feedback could we send to help this person lose weight?” and “What sort of things do you think might get in the way?”

### Self-Monitoring Tools

The usability and acceptability of the self-monitoring tools were explored. First, participants were given an opportunity to use the mFR [57-59]. This image-based dietary assessment tool uses the integrated camera in a smartphone to capture images of food and beverages*.* The images are automatically uploaded to a server for dietary analysis by the research dietitian and used to inform the tailored dietary feedback. The usability and acceptability of the mFR to monitor dietary intake and a wearable device to monitor PA were explored.

### Behavior Change Beliefs Regarding Weight Loss

**Figure 1:**
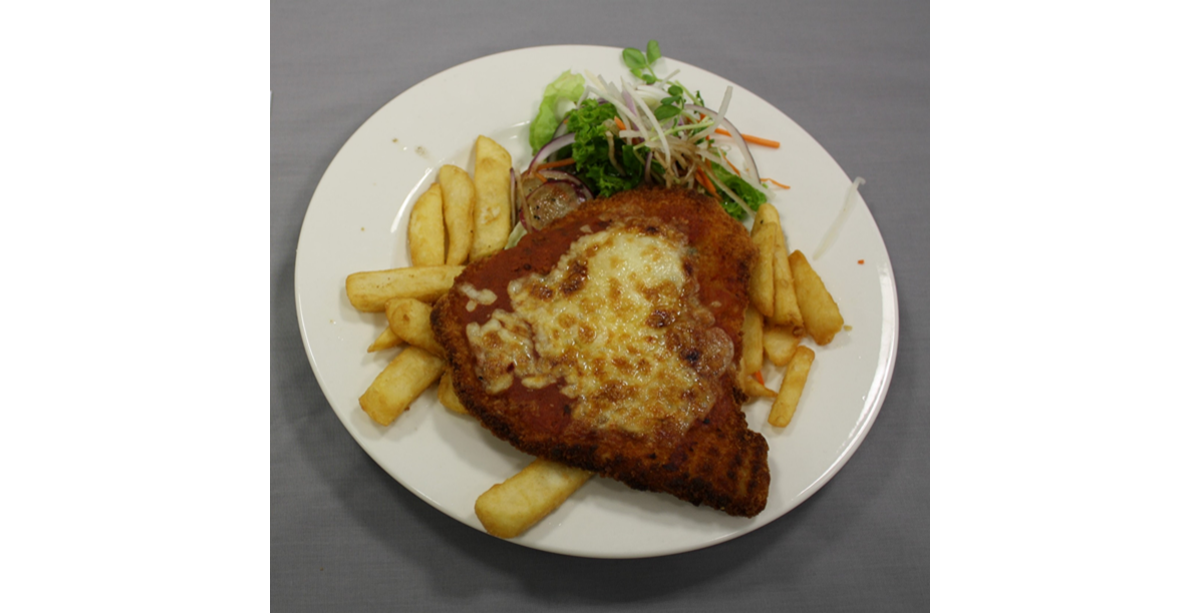
Example of image shown to participants where they were asked what advice they would give this person to help them lose weight.

Participants were given examples of a scenario and asked to provide advice to a hypothetical person to help them lose weight. For example, “people in this study will use an app on their phone to take pictures of their food and drink. Imagine we received this picture from a man wanting to lose weight, what advice should we give him?” (Figure 1). Participants, who were interviewed on the phone, were emailed this information and the images in advance.

### Acceptability of the Feedback Messages

**Figure 2:**
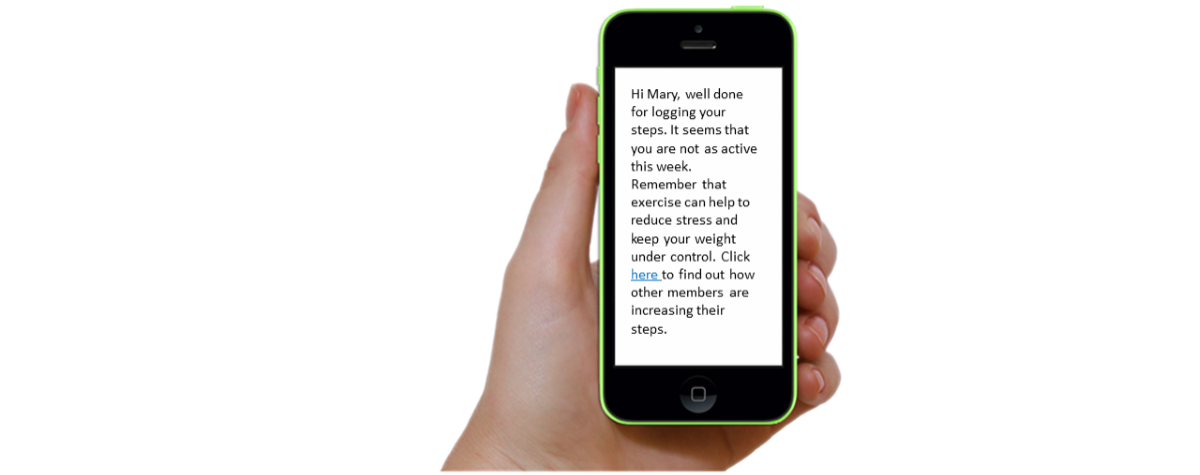
Example of physical activity feedback shown to participants for their comment.

The acceptability of feedback messages, including the length, content, and tone, was explored. Example feedback on diet and PA behavior were shown to participants with questions to explore their understanding and acceptability of the feedback. For example, Figure 2 shows an example of PA feedback where participants were asked to “imagine we sent a person this feedback. How do think they might feel about receiving this feedback? Is there any other information you would add?”

**Figure 3:**
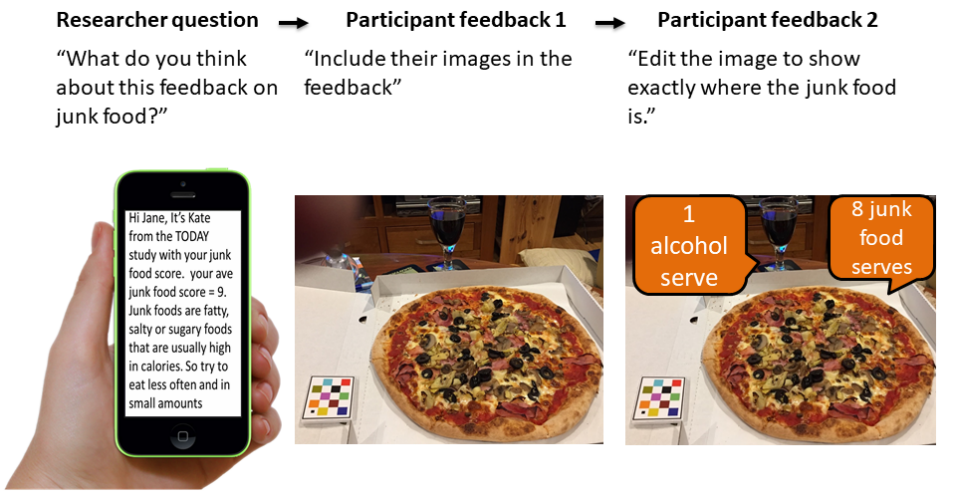
Example images showing how new ideas on how to provide dietary feedback were incorporated using an iterative process based on feedback from the previous focus group and interviews.

After each focus group, new ideas were discussed with the research team (qualitative researcher, dietitian, exercise physiologist, and health psychologist) and potential digital intervention features were developed. The script was adapted after each session using an iterative process to incorporate participant ideas and feedback, which were then explored in the subsequent sessions. Figure 3 provides an example of how intervention features evolved using feedback from participants.

### Data Collection

All interviews were conducted over telephone by author 1, a dietitian (CS; female) who has qualitative research experience [60], with guidance from AB, an established qualitative researcher [61,62]. Participants in the interview completed a consent form and a demographics questionnaire on the web. The focus group participants completed a consent form and a demographic questionnaire on arrival. JH facilitated the male-only group, with CS as a cofacilitator. CS facilitated all other groups, with 1 assistant moderator. Before the interviews and focus groups, participants had no relationship with the researchers and knew the study was about helping to develop a digital weight management intervention.

All focus groups and interviews started with an overview of the proposed ToDAy intervention, where participants monitor their PA with a wearable tracker and record their food and beverage intake with the mFR app. This information was used to provide feedback to help them lose weight. The first activity was a chance to employ the mFR used to capture images of food and beverage intake [57,58]. The facilitator demonstrated how to use the app to take pictures of plastic food models. Participants were then given an opportunity to use the mFR on a mobile device. This exercise served as an icebreaker as well as capturing the questions and comments of participants using the app. This was followed by open-ended questions to start the discussion. Focus groups and interviews lasted between 34 and 78 min and were conducted until reaching a saturation of ideas. At the end of each focus group, the facilitator summarized the main ideas or themes that participants had raised in that group and gained agreement from participants.

### Data Analysis

All audio recordings were professionally transcribed verbatim and reviewed for accuracy by the first author and managed in NVivo. As this study used scripts that evolved between groups, the analysis used a thematic analysis to analyze and code data through the lens of the COM-B model [49]. Qualitative data were analyzed in 3 stages. Following the process of thematic analysis, first was familiarization through reading each transcript, highlighting key points, and discussing the findings with the cofacilitators [63]. The first author led the analysis and developed themes aligning with capability, opportunity, and motivation. The cofacilitators for the focus groups then independently reviewed the scripts. Finally, any discrepancies were reviewed and discussed by the first author. The quotes were then aligned to the final themes [64].

## Results

### Participant Characteristics

Table 2 provides an overview of the characteristics of the participants. A total of 56 adults (32 female and 24 male) from Western Australia participated in 6 telephone interviews and 6 focus groups (average of 5 per group). Of these, over one-third had a BMI>30. Half were employed full time (52%), and most were aged between 25 and 40 years (61%). All participants owned a smartphone (iPhone or Android) and had some experience using apps.

Feedback from focus groups and interviews provided important insights into the acceptability and comprehension of tailored feedback messages. For example, feedback suggesting healthier alternatives to junk food was rejected as these options may not be available and could not be tailored to the individual’s preferences.

**Table 2:** Characteristics of interview participants (N=56).

Characteristics		Values, n (%)
**Age (years)**		
	25-40	34 (61)
	41-65	17 (30)
	>65	5 (9)
**Gender**		
	Male	24 (43)
	Female	32 (57)
**BMI (kg/m^2^)**		
	<25	10 (18)
	25-30	23 (41)
	>30	23 (41)
**Ethnicity**		
	Australian	47 (84)
	Indigenous Australian	5 (10)
	Asian	4 (7)
**Employment status**		
	Employed full time or part time	39 (70)
	Unemployed	8 (14)
	Retired	9 (16)
**Household income Aus** **$ (US $)**		
	<$50,000 (<$35,695)	21 (38)
	$50,000-$150,000 ($35,695-$107,085)	22 (40)
	>$150,000 (>$107,085)	13 (22)

### Qualitative Analysis

Emerging qualitative themes and subthemes were mapped to the COM-B domains. This helped to identify that participants needed support in all 3 areas of capability, opportunity, and motivation.

#### Capability: Misinformation

Capability refers to knowledge and skills related to behavior [45]. Participants’ knowledge of weight loss behaviors was examined by asking them to provide dietary advice to a hypothetical client based on a picture of their food and drink. Responses were themed as misinformation when they provided inaccurate information or nutrition advice. The majority of the discussion focused on giving misinformation as dietary advice. This revealed their knowledge and beliefs about which behaviors are best for weight management. For instance, 2 main examples were discussed. First, potatoes were considered fattening and second, excess alcohol was not a major contributor to weight gain. There was a focus on individual food being responsible for weight gain rather than a holistic view of the total diet.

##### Carbohydrates Cause Weight Gain

Participants were shown an extra-large roast dinner with large meat portions, 3 small potatoes, 3 serves of vegetables, and 6 bottles of beers and asked, “What advice would you give this person to help them lose weight?” All groups commented on the potatoes, only later mentioning the large serve of meat and 6 bottles of beer:

It’s not a healthy meal if it’s got a potato.

Female, FG2

Cut down on the carbs, only 2 potatoes.

Male FG3

##### Alcohol Consumption Does Not Cause Weight Gain

The discussion did eventually focus on reducing the 6 bottles of beer, but there were a number of misinformed views about alcohol situations where 6 beers would be *OK*. For example, if it was a low-carb beer, consumed with fresh lemon, watching football, consumed over the course of an afternoon, the participant has eaten well during the preceding week or has a physical job that would work off excess energy. Some participants were dismissive of the kilojoule content of alcoholic drinks but aware that alcohol may lead to choosing discretionary foods:

So the kebab or the burger is a lot more appealing after a few drinks than it may be if say, while sober.

Male, PI 1

It’s not the beer itself that’s the problem, it’s the food you have with it.

Female, FG2

#### Opportunity: Environmental Support and Social Norms

The *opportunity* component of COM-B relates to physical opportunities, such as the environment and availability as well as social opportunities, including social influences. Both emerged as important themes in the data.

##### Lack of Environmental Support

Environmental factors were discussed as the main barriers to avoiding junk food. One group, in particular, expressed frustration at the density of fast food outlets and the promotion of very low-cost meals that would appeal to children and those on a low income:

When I first came in 1982 there was hardly any fast food places. Now you’ve got 8 or 9 different ones all down the road from each other. Kids have got 2 dollars where are they going to go? For a $2 burger.

Male, FG 5

Participants expressed that the social marketing campaigns and interventions for individuals were futile without addressing the food environment:

…that goes back to the government again because the government has put no restrictions on how much fast food can be in local vicinities. If you look at where they are, it’s not in the high-class areas it’s in the low socio-economic suburbs.

Male, FG 5

##### Unhealthy Food Is Everywhere

Many participants thought that most food options purchased outside the home were not healthy and the serving sizes were too large. Some mentioned added fat, sugar, and salt. Others noted that savory meals often come with chips and sweets are served with cream or ice cream:

When you buy food out... It all tends to be fattening.

Female, FG 2

Most groups brought up opportunistic eating as a key facilitator of eating discretionary food. Events such as at sporting occasions, bake sales, and *sausage sizzles* were given as common examples:

You can’t buy a healthier option at a sausage sizzle, they only have sausage, white bread, and sauce.

Male, FG 4

##### Social Norms

There has been some discussion about the difficulty of social opportunities. Most participants agreed that the expectations of consuming junk food at social occasions were problematic. When eating out, both men and women agreed that it is not socially acceptable for men to ask for healthy options or modification to their order. Regarding swapping chips for vegetables, a man said:

It’s not seen to be manly to be seen like, eating vegetables.

Male, FG 3

If it comes with chips, I’ll eat chips.

Male, FG 4

When discussing ways to reduce alcohol consumption, participants suggested practical methods, such as alternate alcohol with sugar-free soft drinks or choosing a low-strength beer. However, there was an overwhelming consensus that these suggestions were unrealistic and not socially acceptable for men:

…might be difficult if you’re down at the club or in the pub with the guys and then you really get the Mickey taken out of you

Male, FG4

You could suggest having water for every other drink, but who is going to listen to that?

Male, FG 5

#### Motivation

The main theme that emerged from the motivation domain of COM-B was confidence.

##### Confidence

Participants were uncertain about their knowledge or beliefs about food, PA, or weight management. Some of this was linked to the perception that the guidelines from professionals were *always changing*. There was a sense of complacency about the need to or the importance of achieving health recommendations:

What would have been recognized as a healthy meal years ago, these days it’s not a healthy meal because it's got potato; carbs.

Male, PI 3

##### Already Doing Enough

In relation to PA, most adults felt they were already active with daily activities, including looking after children, gardening, housework, and those in nonsedentary employment (eg, nurse, carpenter, and plumber). Motivation to engage in PA was limited by the belief that their lives were already active and busy:

... A mother of 3, busy all day, being told to go for a 45-minute walk. They can’t otherwise they would have done it.

Female FG 2

Concerning eating, most agreed that there was a place for junk foods, namely takeaways, confectionery, and desserts. Making healthy food choices was said to be important, especially for those with health problems like diabetes and high blood pressure. There was a variety of beliefs about how often people should make healthy food choices. Some used broad terms, such as sometimes or not too often. Others believed that healthy eating and being active were part of the working week and not applicable on the weekend:

I mean you can eat healthy but every now and again you can always have take away. But not every week or every day. If you know what I mean?

Female FG 1

During the week it’s structured. You’ve got, you know, time to get to work and your lunch break at work or whatever and then, you know, you come home and it's dinner time and then that's that. Then your weekends are your time to just flop. It’s like a treat. Weekends are your time to relax and enjoy life I guess.

Female, PI 2

##### Reality of Change

When the group was shown potential feedback to address alcohol intake, there was general agreement that changing would be difficult or even impossible. Specifically, several people thought that any feedback to reduce alcohol intake would be futile:

You could tell him to stop at four beers, but by then he won’t know what he’s doing.

Male, FG 5

##### Ambivalence

There was a conflict in the expectations of the interventions. Some felt that feedback should tell them the negative consequences of their poor health choices:

In three years at that rate, your liver is going to look like this.

Male, FG 5

However, feeling judged or reprimanded was cited as a barrier to keeping people engaged and honest about self-reporting. Participants wanted a specific example of food they could swap rather than a general *avoid this* or *chose a healthier option*. At the same time, they said specific advice would be unrealistic:

All well and good to suggest a healthy option but you can’t get your chicken parmigiana grilled with a baked potato at my local, it comes deep-fried with chips.

Female, FG 3

These comments reflect the ambivalence about eating junk food outside the home; something they want help to avoid because it is expensive, is unhealthy, and leads to overeating but something they do because it is convenient and enjoyable.

### Functions and Features of the Intervention

Results from the focus groups and interviews informed the selection of user preferences, intervention functions, and acceptability of the intervention. Table 3 shows the results on the acceptability of digital tools proposed for self-monitoring diet and PA. Participants found the mFR app to be intuitive and convenient in comparison with other digital tools or paper-based methods. All participants shared experiences of using a pedometer and saw the option for a wearable PA tracker as an incentive to join the study. Feedback on the clinical aims highlighted gaps in their understanding of the guidelines for diet and PA. Participants agreed that personalized feedback would promote health-enhancing habits by enhancing confidence and motivation.

The focus groups and interview data were reviewed for 3 intervention functions expected to mediate a behavior change, for example, education, modeling behavior, and persuasive communication.

1. *Education* (to increase knowledge on how to identify EDNP food and on PA guidelines)
2. *Modeling behavior* (annotating food and beverage images by feedback on intake)
3. *Persuasive communication* (images and motivation enhancement using positive reinforcement in tailored communications)

**Table 3:** Understanding user perspectives and experiences of participants on the clinical aims using the mobile food record app and their experiences or views about using a physical activity monitor.

Clinical aims and examples of questions or activity		Participant quotes
**Self-monitor diet**		
	Practice using the mobile food record app	“It’s really intuitive and easy; better than the apps where you need to find the food”
	How easy/difficult would it be to use to capture all your food and drink for 4 days?	“I wouldn’t use it if I was at the club with the guys. I wouldn’t use it at work (nurse)”
**Self-monitor physical activity**		
	Have you ever used an activity tracker? Prompt for wearable device, pedometer, mobile app	“I used to use a watch that tracked steps and heart rate, it was good at first then all the alerts got annoying”
	Any advice or support that helped?	“Yeah it’s good to see that you’ve done like ten thousand steps in a day”
**Increase fruit and vegetable consumption**		
	How much fruit and vegetables are recommended each day?	“2 fruit and 5 veg but I’m not sure if it has to be 5 different types”
	What advice would you give this person to help them lose weight? (Shown example meal)	“your vegetables are supposed to be half your plate so many people don't know that”
	What type of feedback could we send to help this person to get them to eat more vegetables?	“If someone sends you a picture, send it back saying ‘that’s at least one serve of your five today’”
	What sort of things might get in the way?	“No one eats that much veg, it’s impossible”
**Reduce EDNP^a^**		
	What advice would you give this person, to help them lose weight?	“because you’ve eaten this you have to run ten kilometers to work it off, sort of thing”
	What sort of things might get in the way?	“…you go to Bunnings (national hardware chain with fundraising barbecues) you’re going to get your sausage, there’s no other options”
**Reduce intake of alcohol**		
	What advice would you give this person, to help them lose weight?	“Would be much better if he switched to a lighter beer, like only 4%”
	What sort of things might get in the way?	“If you’re out, say watching sport in the afternoon, everyone else is drinking it would be hard to have water”
**Increase active minutes/decrease sedentary behavior**		
	How much physical activity is recommended each day?	“The adverts tell you half an hour a day and ten thousand steps. You can’t do ten thousand steps in half an hour, it doesn’t make sense”
	Any ideas of how we could support them to do this?	“Show it like your bank account where you can track it and see where it’s going”
	What might make it easier/difficult?	“It needs to be friendly and informative and helpful”

^a^EDNP: energy-dense nutrient-poor.

Table 4 summarizes the stages of applying COM-B to the focus groups and interview data to identify nutrition, PA, and weight management intervention functions and features.

New ideas from participants were explored for feasibility and consistency with evidence-based diet and PA guidelines [42,43]. Rejected ideas included individual assessment of vitamin and mineral status and details on how much weight could be lost with a specific number of steps, as this was not consistent with evidence-based advice. Accepted ideas were the inclusion of participant food and beverage images from the mFR into the tailored dietary feedback. For PA, accepted ideas included using graphs for self-comparison of PA levels throughout the study, positive re-enforcement to acknowledge improvements in activity, and regular goal setting. There was a strong preference for visual feedback for both diet and PA. This was not feasible with text messages; therefore, feedback was primarily provided by email. Digital intervention features may continue to develop during the intervention and include aspects that were not originally mapped in this development phase [40].

**Table 4:** Summary of stages of applying capability, opportunity, motivation, and behavior framework to the focus groups and interview data to identify nutrition, physical activity, and weight management intervention functions and features.

COM-B^a^ and themes		User preferences		Intervention functions		Intervention features	
**Capability: psychological**							
	Misinformation		Simple expert advice; links to further information		Education; tailored feedback on performance		Mobile food record; dietary goals
**Capability: physical**							
	Environmental support		Dietary self-monitoring tools is easy, quick, and subtle		Training: how to use the mobile food record app		Mobile food record
	Environmental support		PA monitor is easy to use and is visually appealing		Training: how to use the Fitbit charge 2		Fitbit Charge 2; tailored movement goals
**Opportunity: physical**							
	Environmental support		Feedback provides visual information, basis their food choice		Education: link to Australian guidelines for diet and PA^b^		Tailored dietary feedback
**Opportunity: social**							
	Social norms		Feedback is nonjudgmental, supportive tone		Supportive and friendly tone		Tailored feedback, tailored education
**Motivation**							
	Confidence		Goals for diet, PA, and weight change are realistic goals		Modelling; personalized examples of how to improve their current behavior		Tailored feedback; Fitbit Charge 2; tailored movement goals

^a^COM-B: capability, opportunity, motivation, and behavior.

^b^PA: physical activity.

## Discussion

### Principal Findings

This study reveals a lack of knowledge and confidence about evidence-based weight management behaviors and susceptibility to misinformation about nutrition. The results also suggest that the BCTs of self-monitoring and feedback on performance are well suited to this group. This paper follows previous examples of combining theory and the experience of users and experts to develop digital interventions [47]. Our findings explored factors affecting weight management behavior, all of which could be mapped to the COM-B model. Participants expressed concerns about their capability; misinformation and opportunities; availability of alternatives and motivation or plans to change their behavior [45,47].

The capability theme was most notable, with lack of knowledge and misinformation being most prevalent. Despite awareness and positive attitudes toward the LiveLighter campaign, participants were still unaware of how to implement the messages from the campaign into their own lives. Several researchers have identified this as nutrition literacy, the ability to interpret and use nutrition information [65-67]. Misinformation about effective dietary strategies to manage their weight was evident; the energy content of alcohol was underestimated and potatoes were described as *fattening*. This highlights the need for more tailored and specific information that addresses participants’ ability to understand and implement new behaviors. A recent review recommends interventions provide actionable feedback and information on where and when to perform the new behavior [68]. For example, using the mFR to assess a meal image (Figure 1) and then providing actionable feedback such as identifying sources of EDNP foods and providing suggestions for change.

Motivation to change eating habits and reduce alcohol intake was hindered by beliefs, and government guidelines on alcohol were considered unrealistic, a view found previously [69]. Similarly, participants were aware of the recommendations to eat 2 serves of fruit and 5 serves of vegetables, but did not believe this was necessary [70]. This seems to be compounded by limited or inaccurate knowledge of serving sizes and energy content of foods [71].

Ambivalence appeared to be a strong theme, with participants describing their competing desires. Although most participants admitted to actively try to restrict their intake of junk food, they also revealed that junk foods were associated with happiness and *time off*. Similarly, eating out was viewed as a major barrier to weight management because of excess portion sizes and lack of healthy options. However, it was also described as a compulsory, normal part of our culture. These views are supported by a previous study, which highlighted that people felt pressured to eat junk food to participate socially and avoid criticism from their peers [72]. In addition, behavioral science has identified that our habits are the salient drivers of behavior, rather than motivation or intention to change [73]. This suggests that interventions should focus on developing habitual changes and creating healthy options as the default choice [73].

Social factors appear to be both motivators and barriers to healthy eating. Although most people agree that eating a healthy diet is what *should* be done, the social context of eating was associated with alcohol and junk food. Qualitative studies with Australian men aged 18 to 25 years reported that healthy eating was seen as incongruent with the masculine stereotype [44]. Our study confirmed that this view also exists among older men and women.

### Strengths and Limitations

A major strength of the study was the large sample of participants living with overweight and obesity and the inclusion of male and female participants. Although men are more likely to have a poor diet, carry excess weight, and experience weight-related disease, they are underrepresented in weight loss interventions. As a result, this study sought to recruit male participants to ensure that their views and preferences were represented in the development of the intervention. The transcripts were coded by gender. However, no apparent differences in preferences or views were found between men and women. Further studies with this cohort would benefit from exploring specific gender differences in the experience of weight loss behaviors and gender preferences for digital intervention features.

This research explored digital technology on several platforms, including the mFR app, wearable PA trackers, email, and text messaging. This made it difficult to apply a single framework exclusively for each of these elements to the intervention. As a result, Australian guidelines for weight management, diet, and physical activity informed target behaviors, rather than a theory-based process such as the behavior change wheel, intervention mapping, or the Integrate Design Assess and Share framework [45,74,75].

The use of self-reported height and weight measures was a limitation of the study and may have led to inaccuracies in the reported BMI. At screening, volunteers were excluded if their BMI was <25. However, when asked at the focus groups to self-report their height and weight, 10 participants reported a BMI of <25. Therefore, the views expressed in these focus groups may not entirely reflect those living with overweight and obesity. Study participants primarily registered on the web via the LiveLighter website and were likely to already have an interest in digital weight management. Their preferences may differ from those who have not previously attempted to seek weight loss information online.

A limitation of the study is that the predetermined intention to develop a scalable digital behavior change intervention could have likely restricted the themes that emerged from the data. The purpose of this focus group study was to explore which intervention features would be acceptable and feasible to assist participants’ behavior change. The study explored potential barriers and benefits of using technology, rather than the wider context of the lived experience of participants in relation to their weight issues, such as social support. This is a limitation of the study, and further in-depth research is needed to explore this issue. The strength of this research is that it explored participants’ opinions on a variety of relatively accessible technological devices to gauge their suitability for intervention. Theoretically, these PA trackers are effective and ideal for hard-to-reach groups. Although they provide objective feedback, little is known about users’ experiences or preferences regarding the use of these tools for self-monitoring purposes. Another limitation was that the discussion regarding social support was limited to identifying the desired frequency of researcher contact. This script focused specifically on the supportive features of the intervention and did not explore other social support as they were outside the scope of the study. A further limitation was that the pace at which this intervention was developed and evaluated was protracted in comparison with commercial interventions [76]. Recent industry and academic partnerships have demonstrated the potential to produce high-quality digital interventions at a commercial pace [77].

### Future Directions

Dichotomous thinking about food and activity can impede efforts to make healthy lifestyle changes [78]. A more flexible and nonjudgmental approach can lead to better behavior change and reduce dietary restraint when supporting the psychological well-being of participants [39,79]. This intervention aims to adopt this flexible and nonjudgmental tone. The effectiveness of these strategies will be evaluated in a randomized controlled trial and exit interviews. Future studies will assess the relationship between behavior change and intervention features, consistent with guidelines for developing digital health interventions [49].

### Conclusions

The ToDAy study was developed using a person-centered approach and behavior change theory. Focus groups and interviews were undertaken to explore user capability, opportunity, and motivation to perform the targeted behaviors for weight loss. The study revealed a lack of knowledge, confidence, and susceptibility to misinformation about evidence-based weight management behaviors. The findings suggest that a digital weight management intervention using mobile food records and activity trackers to inform tailored feedback may be an acceptable, feasible, and engaging strategy.

## Appendix

Multimedia Appendix 1 Script for interviews and focus groups.

## Abbreviations

BCT: behavior change technique
BIT: behavioral intervention technology
COM-B: capability, opportunity, motivation, and behavior framework
EDNP: energy-dense nutrient-poor
ToDAy: Tailored Diet and Activity
PA: physical activity
SSB: sugar-sweetened beverage
