# Supplementary material for: Design and Development of a Digital Weight Management Intervention (ToDAy): Qualitative Study
Source: JMIR Mhealth Uhealth. 2020 Sep 9;8(9):e17919. doi: 10.2196/17919 (PMC7511863; doi:10.2196/17919)
Supplement: Multimedia Appendix 1 [file mhealth_v8i9e17919_app1.docx]

## Appendix 1

**Script for interviews and focus groups**

**Ice Breaker**

1. Use the iPad to take a photo of this food (plastic food or real food provided).

**Online weight management**

1. What do you think about weight loss support on your mobile phone or on the internet?
2. If you were going to design an online weight management program what kinds of things would you include?
3. What do you think would encourage you to stay engaged with an online weight management program?

**Mobile food record app**

1. We have an app to help people monitor their food and drink. You take a photo of your food/drink before and after you eat or drink. The app automatically sends us the photos with the time and date. This allows us to send personalized feedback to help with weight loss. Use the iPad to have a go at taking a picture of this apple?
2. Imagine you were asked to use this app. What might you like/dislike? How long would you be willing to use it for?

**Advice to help people lose weight (slides shown in this section evolved based on feedback from previous focus groups and interviews)**

1. Have a look at this picture (examples included large portion, junk food, excess alcohol and physical inactivity). What advice do you think we should give this person to help them lose weight?
2. Have a look at the feedback we plan to send
   1. How would you rate this message?
   2. Who do you think the message should come from?
   3. Is there any other information you would like?
   4. Can you suggest any support to help make this change?
   5. Can you think of anything that might get in the way?
   6. How easy do think it would be to maintain this new behavior?

**Example questions on target behaviors (junk food, portion sizes, alcohol and physical activity)**

1. If you were getting feedback about how to (change x behavior); what type of information would be helpful?

b. The last group suggested that dietary feedback include the participants own food images in their feedback so they could see where the junk food came from. What do you think of this idea? Have a look at this example. Is there anything you would change?

**Engagement and support**

1. Imagine you have been selected to take part in this study.
   1. What sort of messages or support would you find helpful?
   2. Is there anything that you would not like?
   3. Another group suggested we sent testimonials from other participants. What do you think of this idea?
   4. Another group suggested we send links to online resources and further information. What do you think of this idea?
